# *Usnea* improves high-fat diet- and vitamin D3-induced atherosclerosis in rats by remodeling intestinal flora homeostasis

**DOI:** 10.3389/fphar.2022.1064872

**Published:** 2022-11-25

**Authors:** Yanjun Liu, Chongyang Dou, Guihua Wei, Liudai Zhang, Wei Xiong, Lingmiao Wen, Chunxiao Xiang, Chunlan Chen, Tinglan Zhang, Alvin Altamirano, Yunhui Chen, Tian-e Zhang, Zhiyong Yan

**Affiliations:** ^1^ School of Life Science and Engineering, Southwest Jiaotong University, Chengdu, China; ^2^ Department of Chemistry and Biochemistry, Northern Arizona University, Flagstaff, AZ, United States; ^3^ School of Basic Medicine, Chengdu University of Traditional Chinese Medicine, Chengdu, China

**Keywords:** *Usnea*, atherosclerosis, intestinal flora, lipid metabolism, inflammatory response

## Abstract

**Background:** Usnea has various pharmacological properties, including anti-inflammatory, antitumor, antioxidant, antiviral, and cardiovasculoprotective effects.

**Aim of the study:** To investigate the potential mechanisms underlying the anti-atherosclerosis (AS) activity of *Usnea* ethanol extract (UEE) *via* the regulation of intestinal flora.

**Materials and Methods:** The chemical composition of UEE was determined using ultra-performance liquid chromatography with quadrupole exactive orbitrap mass spectrometry (UPLC-Q-EOMS). Thirty-six male Sprague–Dawley rats were divided into six groups. A high-fat diet and intraperitoneal vitamin D3 injections were used to establish a rat model of AS. After 4 weeks of treatment with UEE, hematoxylin–eosin staining was performed to evaluate the pathomorphology of the aorta, liver, and colon. The composition and diversity of the rat intestinal flora were determined using high-throughput 16S rRNA sequencing. Enzyme-linked immunosorbent assays were used to measure the levels of plasma trimethylamine oxide (TMAO), serum bile acid (BA), total cholesterol (TC), triglyceride (TG), high-density lipoprotein cholesterol (HDL-C), low-density lipoprotein cholesterol (LDL-C), lipopolysaccharide (LPS), tumor necrosis factor-alpha (TNF-α), and interleukin-6 (IL-6). The protein expression of cholesterol 7α-hydroxylase (CYP7A1) and flavin monooxygenase 3 (FMO3) in the liver and zonula occludens-1 (ZO-1) and occludin in colon tissue was detected *via* western blotting.

**Results:** Forty-four compounds were identified in UEE. In the rat model of AS, UEE significantly prevented calcium deposition; decreased the serum levels of TC, TG, LDL-C, LPS, TNF-α, and IL-6; and increased the serum level of HDL-C. Additionally, all UEE dosages decreased the relative abundance of *Verrucomicrobiota* while increased that of *Bacteroidetes*. FMO3 protein expression and TMAO levels decreased, whereas CYP7A1 protein expression and BA levels increased. The absorption of intestinal-derived LPS was minimized. Furthermore, the protein expression of ZO-1 and occludin was upregulated.

**Conclusion:** UEE ameliorated AS. The underlying mechanism was the reversal of imbalances in the intestinal flora by *Usnea*, thereby inhibiting calcium deposition, abnormal lipid metabolism, and inflammatory response.

## 1 Introduction

Atherosclerosis (AS) is a chronic inflammatory disease that is characterized by aberrant lipid metabolism, macrophage foaminess, calcification, and plaque deposition (Libby, 2021). Statins, fibrates, and other drugs are now used in clinical trials to treat AS (Zhao et al., 2019). However, the long-time use of lipid-lowering medications is associated with withdrawal symptoms, gastrointestinal responses, and allergic reactions (Florentin et al., 2011). Consequently, the treatment of AS necessitates the exploration of novel therapeutic pathways and the discovery of new effective medications. Diets that are high in fat can affect the composition and function of intestinal flora over time (Schoeler and Caesar, 2019). Additionally, there is a causal link between intestinal flora and cardiovascular disease (Marques et al., 2017). Alterations in the composition and function of intestinal flora can result in abnormalities in lipid metabolism and intestinal inflammation, which ultimately contribute to the development and progression of AS *via* metabolic and nonmetabolic (inflammatory) pathways (Navab-Moghadam et al., 2017).

With regard to metabolic pathways, the results of many clinical studies have shown that excessive amounts of total cholesterol (TC), triglycerides (TGs), and lipoproteins in the blood are indicative of aberrant lipid metabolism and a major cause of the development and progression of AS (Albertini et al., 2002). The conversion of cholesterol to bile acids (BAs) is dependent on the body’s reverse cholesterol transport (RCT) (Jessup et al., 2006). In the presence of intestinal flora, primary BAs are converted into secondary BAs *via* dehydrogenation and dehydroxylation performed by this flora (Jessup et al., 2006). Trimethylamine oxide (TMAO) inhibits the activity of several rate-limiting enzymes (e.g., cholesterol 7α-hydroxylase [CYP7A1] and sterol 27-hydroxylase), decreases the production of primary BAs, and impairs the RCT process, which causes a decrease in cholesterol efflux; this results in marked accumulation of cholesterol in tissue cells and promotes the formation of phagocyte-derived foam cells (Koeth et al., 2013). Methylamine-containing substances (such as choline and L-carnitine) can be converted to trimethylamine by intestinal flora, which then enters the liver *via* the blood circulation and is oxidized to TMAO by hepatic flavin-containing monooxygenase (FMO). Dietary supplementation with TMAO can elicit inexplicable AS in mice (Wang et al., 2011). FMO3 is the principal protease in the oxidation of TMAO and is the most active member of the FMO family of proteins (Bennett et al., 2013).

Regarding nonmetabolic pathways, the long-term consumption of a high-fat diet causes an increase in the concentration of lipopolysaccharide (LPS) in the body; activation of toll-like receptor 4 in the intestine; excessive release of inflammatory factors, such as tumor necrosis factor-alpha (TNF-α), interleukin (IL)-6, and IL-1β; and a subsequent local inflammatory response in the intestine (Suzuki et al., 1997). When prolonged local inflammation affects intestinal permeability, LPS and microbes enter the body’s circulation through the injured intestinal epithelium, thereby triggering or exacerbating AS lesions (Cani et al., 2007; Zhu et al., 2018). The blood levels of TNF-α, IL-1β, and IL-6 can be used for the diagnosis of illnesses and guiding therapy in patients with AS (Woollard, 2013).

*Usnea* is a lichen of *Usnea diffracta* Vain and *Usnea longissima* Ach, which belongs to the genus *Usnea* Adans of Usneaceae. “*Usnea* is bitter in taste and has mild nature, it control anger and evil qi, it can stop abnormal sweating due to general debility and head wind”, according to the *Shen Nong’s Herbal Classic*, where it was firstly mentioned (Gu, 1955). Zhao et al. found that *Usnea* extract could increase lipid metabolism in AS rat model through TLR5/NF-κB signaling pathway and reduce the aortic plaque area (Zhao et al., 2019). Zhang et al., under the guidance of the TCM theory of “Exterior-Interior Relationship between Heart and Small Intestine”, found that *Usnea* could treat AS by improving the disorder of intestinal flora structure, enhancing the intestinal mucosal barrier, and reducing the levels of serum LPS and inflammatory factors (Zhang et al., 2021). *Usnea* has been proven in modern pharmacological investigations to have a variety of pharmacological effects, including anti-inflammatory (Huang et al., 2011), anti-tumor (Yamamoto et al., 1995), antioxidant (Bian et al., 2002), anti-virus (Campanella et al., 2002), and cardiovascular protection (Behera et al., 2012).

In this study, we investigated the effects of AS on colonic intestinal flora as well as the relationship between colonic intestinal flora and lipid metabolism and inflammatory response, utilizing the intestinal flora as a target to understand the possible mechanism of action of *Usnea* in the treatment of AS.

## 2 Materials and methods

### 2.1 Chemicals and reagents

Wuhan Servicebio Technology Co., Ltd. Provided hematoxylin ([E] ID: G1004) and eosin ([E] ID: G1001). Nanjing Jiancheng Bioengineering Institute (Nanjing, China) supplied the TC assay kit (ID: 20210306), TG assay kit (ID: 20210308), high-density lipoprotein cholesterol (HDL-C) assay kit (ID: 20201205), low-density lipoprotein cholesterol (LDL-C) assay kit (ID: 20201208), and BAs (ID: 20201224). Shanghai Fankel Biotechnology Co., Ltd. (Shanghai, China) provided the TMAO test kit (ID: Jan-2021). Quanzhou Ruixin Biotech Co., Ltd. (Quanzhou, China) provided the LPS test kit (ID: Dec 2020). The TNF-α assay kit (ID: 22J328) and IL-6 assay kit (ID: 22J288) were purchased from Yikesai Biotechnology Co., Ltd. (Taicang, China). Anti-CYP7A1 rabbit monoclonal antibody (ID: 510615), anti-zonula occludens-1 (ZO-1) rabbit monoclonal antibody (ID: AF5145), anti-occludin rabbit monoclonal antibody (ID: 502601), anti-FMO3 rabbit monoclonal antibody (ID: 389347), anti-β-actin rat antibody (ID: DD1027), goat anti-rabbit IgG (H&L) (ID: JJ0903), and goat anti-rat secondary antibody (ID: II1120) were provided by Chengdu Zen Bioscience Co., Ltd. (Chengdu, China). The BIOMICS DNA microprocessor kit (ID: D4301) and Zymo clean gel recovery kit (ID: D4008) were purchased from Zymo Research (United States). The NEBNext FFPE Repair Mix (ID: M6630) and NEBNext Ultra II End repair/dA-tailing Module (ID: E7546) were purchased from New England Biolabs (United States). The 16S Barcoding Kit (ID: SQK-RAB204), Ligation Sequencing Kit (Lot SQK-LSK109), Flow Cell Priming Kit (ID: EXP-FLP002), and MinION Flow Cell (ID: R9.4) were purchased from Nanopore Inc. (United States).

### 2.2 Plant collection and extraction

*Usnea* was purchased from Chengdu’s Lotus Pond Chinese herbal medicine market. The specimen (no. 2020100101) was kept in the Pharmacological Laboratory of Traditional Chinese Medicine at Southwest Jiaotong University’s School of Life Science and Engineering. The plant name was verified using the Plant List (http://www.theplantlist.org/).

At room temperature, dried *Usnea* (800 g) was extracted twice with six volumes of 80% ethanol for 24 h and four volumes of 80% ethanol for 12 h. After filtering, the extract was concentrated and dried for 48 h to provide about 48 g of UEE. The extraction yield was 6.0%.

### 2.3 Ultra-performance liquid chromatography coupled with quadrupole exactive orbitrap mass spectrometry (UPLC-Q-EOMS) analysis of UEE

The chemical ingredients of UEE were detected using UPLC-Q-EOMS. The chromatography of UEE was performed as previously described (Xiong et al., 2022). The following MS settings were used: ion spray voltage, 3.0 kV for (−)-electrospray ionization (ESI) and 3.5 kV for (+)-ESI; sheath gas flow rate, 30 arbitrary units; auxiliary gas, 7%; ion-transport tube temperature, 320°C; heated vaporizer temperature, 350°C; scanning mode, full MS. Data were acquired and analyzed using Xcalibur 4.0 and Compound Discoverer 3.1. (Thermo Fisher Scientific).

### 2.4 Experimental animal grouping and drug administration

Thirty-six specific-pathogen-free Sprague–Dawley rats (body weight, 170–190 g) were obtained from the Dashuo Biological Technology Company (Chengdu, China) with the license number SCXK (Chuan) 2020–030. All procedures were performed in strict compliance with the relevant regulations of Southwest Jiaotong University and in accordance with the code of ethics for animals (No. SWJTU-2006–001). All rats were randomly divided into six groups. Except for rats in the normal group, all rats received a single intraperitoneal (i.p.) injection of vitamin D3 (VD3) (6 × 105 IU/kg) and were then fed a high-fat diet (3.5% cholesterol, 0.5% sodium cholate, 0.2% propylthiouracil, 5% white sugar, 10% lard, and 80.8% basal diet) for 14 weeks (Cai et al., 2005).

In a previous study, 2.766 g/kg/day of *Usnea* was administered to rats to achieve a good lipid-lowering effect (Zhu et al., 2017). Therefore, in the present study, the *Usnea*_L, *Usnea*_M, and *Usnea*_H groups received 0.7, 1.4, and 2.8 g/kg/day of UEE, respectively. Simvastatin was used as the positive-control drug in this study and administered at a dosage of 0.004 g/kg/day (Zhu et al., 2017). The drugs were administered into each group of rats by gavage once daily for 4 weeks at a gavage volume of 1 mL/100 g.

### 2.5 Sample collection and testing

After the administration of the last dose of the drugs, the rats were anesthetized and blood was collected into nonanticoagulated centrifuge tubes, followed by separation of serum by centrifugation at 3,000 rpm (4°C) for 15 min after allowing to stand at 25°C for 30–60 min. Blood was also collected in sodium heparin-containing centrifuge tubes, and plasma was separated immediately by centrifugation at 3,000 rpm (4°C) for 15 min. After the blood collection was completed, all rats were sacrificed and the aorta, colonic tissue, and colonic fecal samples were collected and stored at −80°C.

### 2.6 Histological analysis

The aortic, liver, and colon tissues were fixed with 4% paraformaldehyde for 48 h, followed by dehydration, embedding in paraffin, sectioning, and H&E staining. Finally, light microscopy was used to examine the pathogenic alterations (the scale of the images is 100 μm).The degree of aortic tissue lesions was scored in four levels, with normal vessels or no atherosclerosis scored as 0, atherosclerosis area ≤ 20% scored as 1, atherosclerosis area between 20% and 50% scored as 2, and atherosclerosis area > 50% scored as 3. The degree of liver and colon tissue lesions was scored on a five-point scale, with no lesions or minimal lesions scored as 0; mild lesions or a small amount of lesions scored as 1; moderate lesions or a moderate amount of lesions scored as 2; severe lesions or a large amount of lesions scored as 3; and very severe lesions or a large amount of lesions scored as 4.

### 2.7 ELISA analysis

TC, TGs, LDL-C, HDL-C, TMAO, BAs, LPS, TNF-α, and IL-6 were quantified using specific kits according to the manufacturers’ instructions.

### 2.8 Western blot assay

After the thoracic aorta, liver, and colon tissue of the rats were weighed, proteins were extracted with liquid nitrogen, ground, dissolved in 10 × tissue lysis fluid, and centrifuged at 13000 rpm for 10 min. Next, proteins were separated using SDS–PAGE and transferred to polyvinylidene fluoride membranes. The primary antibodies (anti-β-actin, anti-FMO3, anti-CYP7A1, anti-ZO-1, and anti-occludin) were incubated with the membrane overnight. Subsequently, the membrane was washed three times with phosphate-buffered saline and incubated with a horseradish peroxidase-conjugated rabbit secondary antibody for 2 h at 25°C. Finally, the protein bands were detected using electrogenerated chemiluminescence and quantified using the ImageJ software.

### 2.9 16S rRNA amplicon sequencing of intestinal microbiota

From each group, three samples were chosen at random for sequencing. The detection process can be described as follows: (1) Sample DNA purification—the sample genomic DNA (gDNA) was purified using the Zymo Research BIOMICS DNA Microprep Kit, and gDNA integrity was detected using 0.8% agarose electrophoresis, followed by nucleic acid concentration detection using Tecan F200. (2) PCR amplification—according to the sequencing region, the full-length 16S rDNA gene fragment of the sample was amplified using specific primers with full-length 16S universal barcode, and the amplified primer sequence was Primer 5′–3': 8F (5′-AGA​GTT​TGA​TCA​TGG​CTC​AG-3′) and 1492R (5′-CGG​TTA​CCT​TGT​TAC​GAC​TT-3′). (3) The PCR products were detected by target fragment electrophoresis using 1% agarose gel. The samples that passed the assay were recovered by taking the target bands using Zymoclean Gel Recovery Kit and mixing them in equimolar amounts after assay quantification. (4) The library was built using the Nanopore R9.4.1 library building kit, and the library was sequenced using Nanopore GridION sequencer and subjected to real-time high-precision base calling.

After removing low-quality sequences and chimeras using tools such as Qcat and NanoFILT we obtained target sequences that were used for subsequent analysis. We used Kraken2 to annotate species and select representative sequences, and then used the species taxonomic database to classify species taxonomic information and construct the operational taxonomic unit (OTU) table. OTUs are a way to cluster similarity. Sequences are clustered, and a large number of sequenced sequences are clustered into a smaller number of taxonomic units, and each OTU provides a representative sequence, based on the subsequent species annotation, and the analysis is easier and clearer. By performing a sequence comparison, appropriate evolutionary models and reconstruction methods are selected to construct evolutionary trees based on the base differences between sequences and sequence characteristics. Community composition analysis, alpha diversity, and beta diversity analysis were performed using R language, and differential species analysis was performed using Python.

### 2.10 Statistical analysis of data

The data generated in this study were statistically analyzed using the SPSS 25.0 software, and the results are expressed as the mean ± standard deviation (X ± SD). Comparisons among multiple samples were performed using one-way analysis of variance, followed by the least significant difference test as a post-hoc test. The Kruskal–Wallis nonparametric test was used to compare differences between different groups of pathological tissues. A *p*-value of < 0.05 was considered statistically significant. Graphs were prepared using the GraphPad Prism 18.0 software.

## 3 Results

### 3.1 Identification of chemical composition of UEE

**FIGURE 1:**
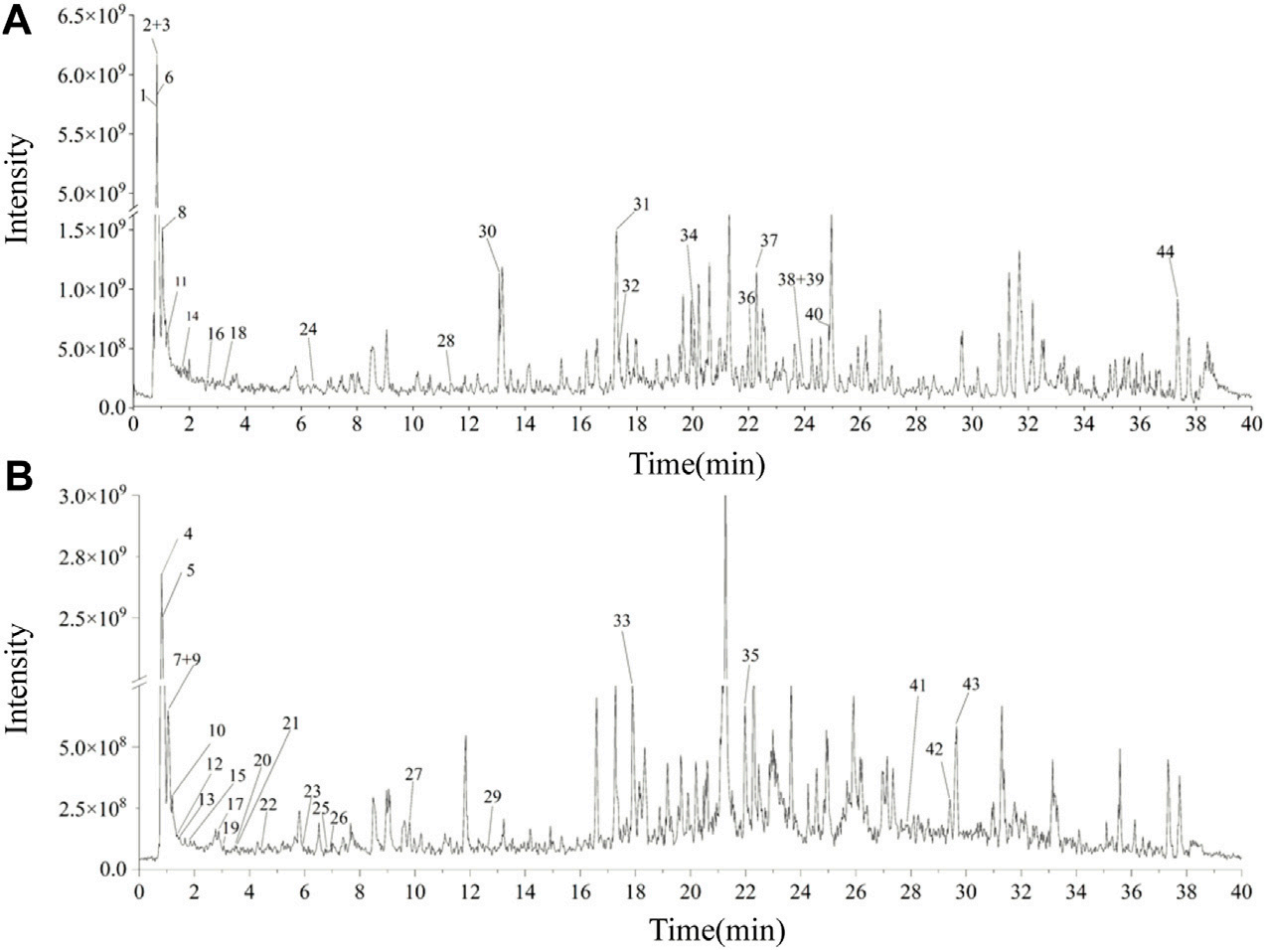
The total ion chromatograms of UEE. **(A)** Total ion chromatograms of UEE in the (+)-ESI. **(B)** Total ion chromatograms of PMC in the (−)-ESI.

Forty-four substances were identified in UEE based on retention time, molecular ions, and MS^2^ spectra by comparison with online (mzCloud) and in-house (mzVault) databases. The order of the top ten response intensity of the mass spectrum was: betaine, citricacid, D-(+)-Arabitol, choline, DL-Norleucine, α-Eleostearicacid, azelaicacid, 6-Hydroxy-8-methoxy-3-methyl-3,4-dihydro-1H-isochromen-1-one, DL-Arginine. Strangely enough, choline or betaine may promotes up-regulation of multiple macrophage scavenger receptors linked to atherosclerosis (Wang et al., 2011), but azelaicacid has obvious anti-atherosclerotic effect (Litvinov et al., 2010). The high-resolution extracted total ion chromatograms of UEE in the (+)-ESI and (−)-ESI modes are shown in Figure 1. Table 1 presents data on the retention time (t_R_), formula, experimental mass, theoretical mass, mass error, MS/MS fragment data, and identified chemicals.

**TABLE 1:** UPLC-Q-EOMS data of 44 characterized compounds in UEE.

No.	tR/min	Identification	Formula	Area	Mode	Experimental Mass	Theoretical Mass	Mass Error(δ)/ppm	mzVaultBest Match	mzCloudBest Match
1	0.774	Choline	C5H13NO	2460917345	[M + H]+	104.10734	104.10699	3.36	83.5	89.2
2	0.842	Betaine	C5H11NO2	13846728026	[M + H]+	118.08656	118.08626	2.58	94.5	88.9
3	0.842	D-(+)-Proline	C5H9NO2	69271875.99	[M + H]+	116.07099	116.07060	3.36	92	81.3
4	0.853	D-(+)-Arabitol	C5H12O5	5739874867	[M-H]-	151.06073	151.06120	−3.09	96.1	87.6
5	0.859	Methylsuccinicacid	C5H8O4	60011551.88	[M-H]-	131.03438	131.03498	−4.58	95.1	81.2
6	0.959	DL-Arginine	C6H14N4O2	381701874.4	[M + H]+	175.11916	175.11895	1.20	95.5	89.9
7	1.035	Nicotinicacid	C6H5NO2	147514151.1	[M-H]-	124.03971	124.03931	3.27	88.2	86.8
8	1.035	2-Hydroxyphenylalanine	C9H11NO3	64561406.89	[M + H]+	182.08156	182.08117	2.14	90.3	90.1
9	1.047	Citricacid	C6H8O7	6540796178	[M-H]-	191.01936	191.01973	−1.94	95.2	85.9
10	1.193	4-Acetamidobutanoicacid	C6H11NO3	161515625.5	[M-H]-	144.06596	144.06662	−4.58	96.9	84.4
11	1.205	DL-Norleucine	C6H13NO2	740701407.3	[M + H]+	132.10223	132.10190	2.50	89.5	83.8
12	1.293	Gallicacid	C7H6O5	11182531.87	[M-H]-	169.01379	169.01425	−2.72	90.6	81.9
13	1.474	Glutaricacid	C5H8O4	11582136.65	[M-H]-	131.03433	131.03498	−4.96	95.4	80
14	1.703	L-Phenylalanine	C9H11NO2	169430683.5	[M + H]+	166.08649	166.08626	1.38	94.3	86.7
15	1.848	2,4-Dihydroxybenzoicacid	C7H6O4	22963356.06	[M-H]-	153.01889	153.01933	−2.89	84.3	85.2
16	2.646	5′-S-Methyl-5′-thioadenosine	C11H15N5O3S	23833783.67	[M + H]+	298.09576	298.09684	−3.62	86.6	88.4
17	2.981	Isophthalicacid	C8H6O4	58465578.59	[M-H]-	165.01891	165.01933	−2.56	82.5	86.8
18	3.267	4-Pyridineaceticacid	C7H7NO2	33289480.88	[M + H]+	138.05524	138.05496	2.03	83.2	84.2
19	3.173	4-Hydroxybenzoicacid	C7H6O3	41250585.99	[M-H]-	137.02383	137.02442	−4.31	89.9	83
20	3.451	Unknown	C16H18O8	13469337.89	[M-H]-	337.09338	337.09289	1.45	91.1	80.9
21	3.581	2-Isopropylmalicacid	C7H12O5	18379886.28	[M-H]-	175.06091	175.06120	−1.66	81	85.5
22	4.334	Pimelicacid	C7H12O4	24915197.04	[M-H]-	159.06580	159.06628	−3.02	87.7	84.6
23	5.922	N-Acetyl-D-alloisoleucine	C8H15NO3	23368451.68	[M-H]-	172.09753	172.09791	−2.21	92.7	84
24	6.534	4-Methoxysalicylicacid	C8H8O4	85613244.22	[M + H]+	169.04974	169.04954	1.21	89.6	90.5
25	6.825	Caffeicacid	C9H8O4	3,0341887.61	[M-H]-	179.03458	179.03498	−2.23	82.9	86
26	6.965	Subericacid	C8H14O4	72710311.41	[M-H]-	173.08140	173.08193	−3.06	89.3	85.3
27	9.805	Azelaicacid	C9H16O4	452817658.5	[M-H]-	187.09720	187.09758	−2.03	82	80.2
28	11.376	L-Ergothioneine	C9H15N3O2S	45961695.95	[M + H]+	230.09529	230.09577	−2.09	90	92.7
29	12.628	3-tert-Butyladipicacid	C10H18O4	33787312.51	[M-H]-	201.11299	201.11323	−1.19	83.8	80.7
30	13.082	(2R)-7-hydroxy-8-(2-hydroxyethyl)-5-methoxy-2-methyl-3,4-dihydro-2H-1-benzopyran-4-one	C13H16O5	44272177.21	[M + H]+	253.10603	253.10705	−4.03	83.1	81.1
31	17.244	(-)-Caryophylleneoxide	C15H24O	29779640.14	[M + H]+	221.18935	221.18999	−2.90	81.3	92.6
32	17.374	6-Hydroxy-8-methoxy-3-methyl-3,4-dihydro-1H-isochromen-1-one	C11H12O4	392430636.4	[M + H]+	209.08058	209.08084	−1.22	87.2	91.2
33	17.875	3-Coumaricacid	C9H8O3	119319093.6	[M-H]-	163.03931	163.04007	−4.66	85	80.5
34	20.1	β-Asarone	C12H16O3	53378148.53	[M + H]+	209.11696	209.11722	−1.24	89.2	96.2
35	22.109	Genistein	C15H10O5	46072326.25	[M-H]-	269.04578	269.04555	0.85	93.1	83.5
36	22.175	Abieticacid	C20H30O2	19321804.16	[M + H]+	303.23041	303.23186	−4.77	89.3	87.7
37	22.291	α-Eleostearicacid	C18H30O2	639904439.8	[M + H]+	279.23065	279.23186	−4.32	87.6	87.3
38	23.965	1,4a-dimethyl-9-oxo-7-(propan-2-yl)-1,2,3,4,4a,9,10,10a-octahydrophenanthrene-1-carboxylicacid	C20H26O3	128520436.2	[M + H]+	315.19394	315.19547	−4.85	87.7	85.8
39	23.99	Nootkatone	C15H22O	11379220.07	[M + H]+	219.17410	219.17434	−1.10	90.4	89.8
40	24.866	Isotretinoin	C20H28O2	61839048.97	[M + H]+	301.21487	301.21621	−4.45	80.2	81.2
41	27.856	Myristylsulfate	C14H30O4S	85748989.62	[M-H]-	293.17972	293.17920	1.77	81.6	86.8
42	29.402	Eicosapentaenoicacid	C20H30O2	40821461.31	[M-H]-	301.21756	301.21730	0.85	83.6	83.9
43	29.636	Pinolenicacid	C18H30O2	272319696.5	[M-H]-	277.21762	277.21730	1.15	84	85.6
44	37.339	α-Linolenicacid	C18H30O2	178313916.1	[M + H]+	279.23047	279.23186	−4.98	85.2	84.2

### 3.2 UEE reverses pathogenic alterations

#### 3.2.1 Aorta pathological changes

**FIGURE 2:**
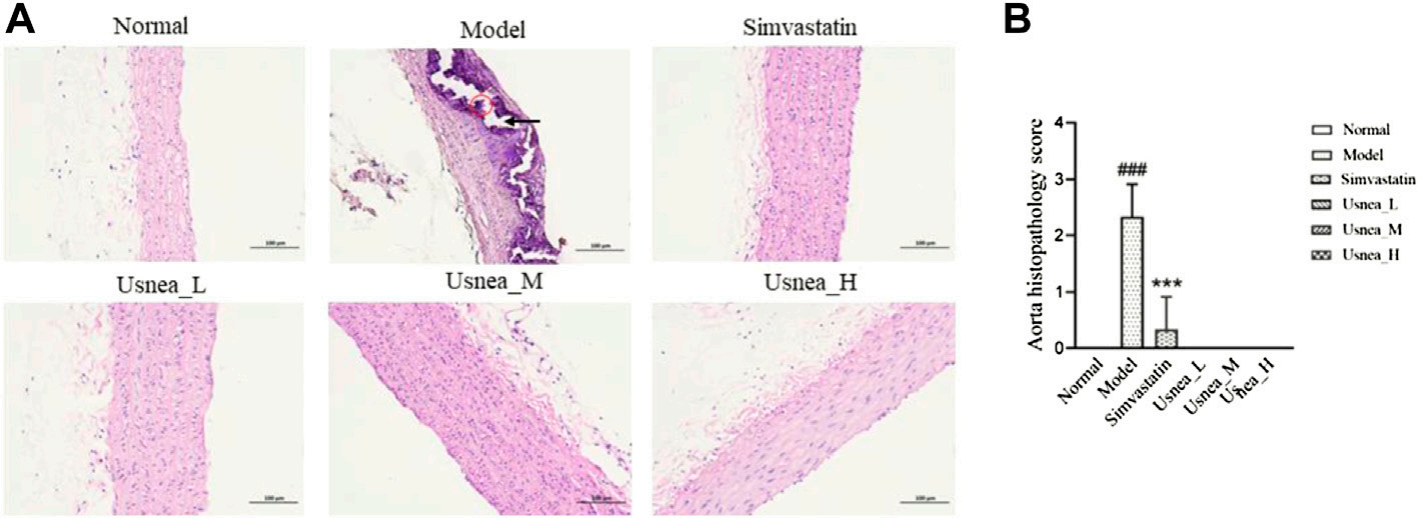
Pathological status of the aorta in each group of rats (200 × ). **(A)** In AS rats, some endothelial cells were missing in the aortic tissue, the endothelium was damaged (black arrow), and the thickness of the middle membrane was uneven; moreover, the nuclei of the smooth muscle cells were irregularly shaped and large calcium salt deposits were visible (red circle). Results are expressed as the mean ± SD (*n* = 6 per group). **(B)** Aorta histopathological score, *n* = 3. Compared with the normal group, ^###^ *p* < 0.001; compared with the model group, ^**^ *p* < 0.01, ^***^ *p* < 0.001.

As shown in Figure 2, The model group rats have obvious calcium salt deposition. In the remaining groups, the aortic tissue of rats had normal aortic morphology, neatly arranged smooth muscle cells, and there were no pathological changes.

#### 3.2.2 Liver pathological changes

**FIGURE 3:**
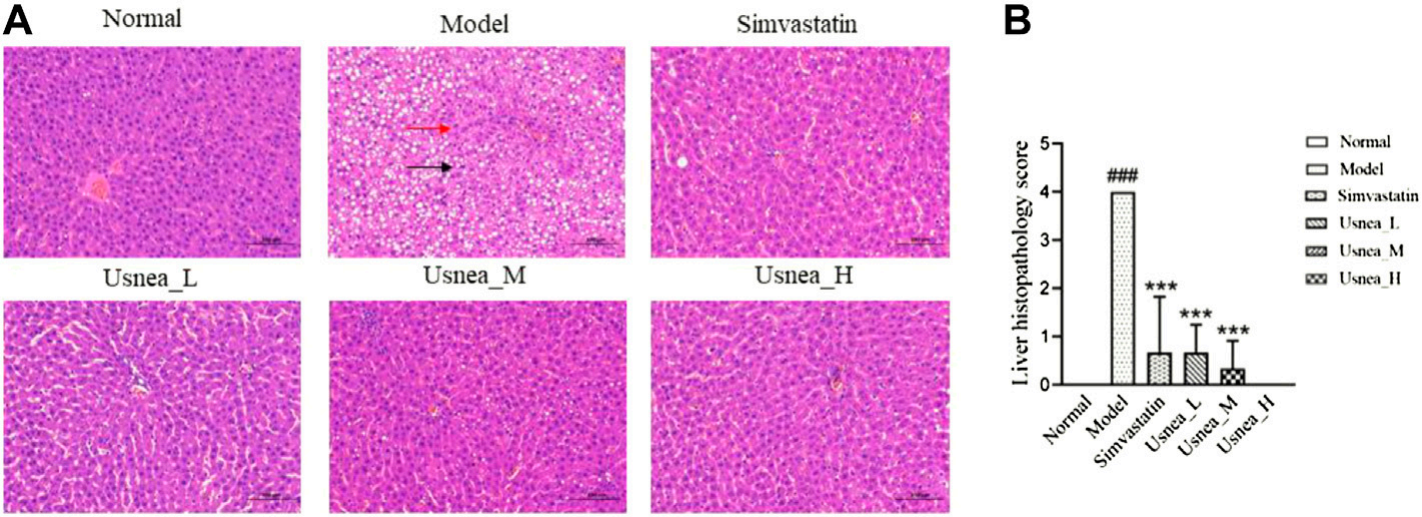
Pathological status of the liver in each group of rats (200 × ). **(A)** In AS rats, the liver exhibited diffuse hepatocyte granular degeneration with a swollen cytosol and a loose and lightly stained cytoplasm (black arrow), a large number of hepatocytes with steatosis, and a variable number of tiny round vacuoles in the cytoplasm (red arrow). Results are expressed as the mean ± SD (*n* = 6 per group). **(B)** Liver histopathological score, *n* = 3. Compared with the normal group, ^###^ *p* < 0.001; compared with the model group, ^**^ *p* < 0.01, ^***^ *p* < 0.001.

As shown in Figure 3, normal rat liver tissue showed no evident pathological alterations. The liver in the model group revealed a large number of hepatocytes with steatosis. A small degree of hepatocyte steatosis and microscopic round vacuoles in the cytoplasm were observed in the simvastatin and *Usnea*_L, *Usnea*_M, and *Usnea*_H groups.

#### 3.2.3 Colon tissue pathological changes

**FIGURE 4:**
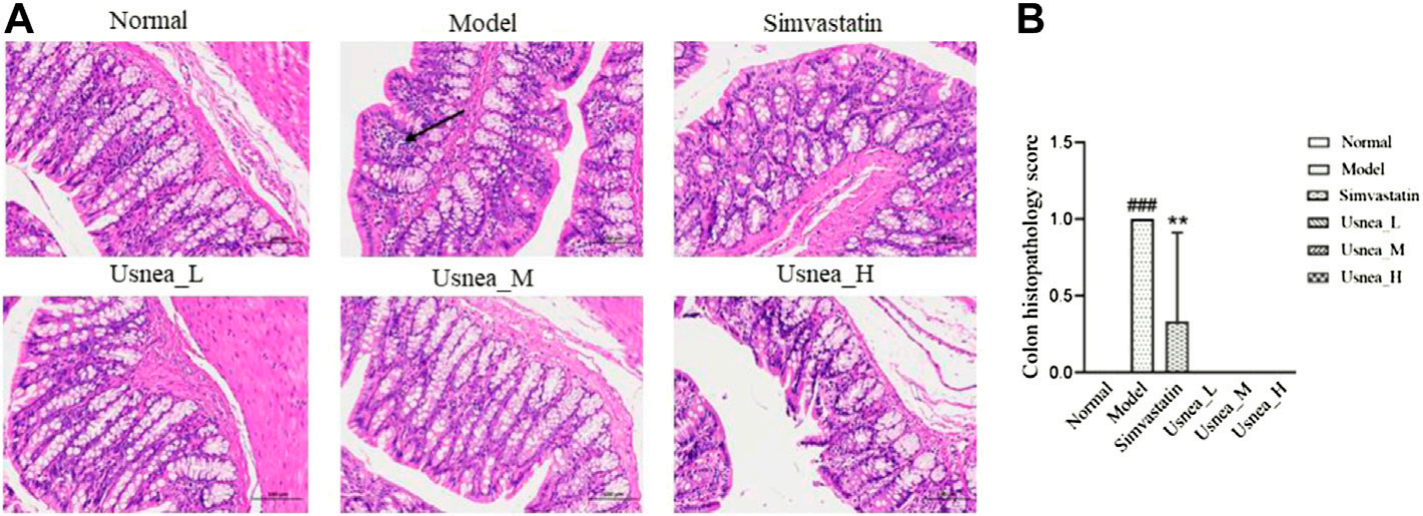
**(A)** Pathological status of the colon tissue in each group of rats (×200). The mucosal layer of colonic tissue was infiltrated by focal inflammatory cells (black arrow). Values are expressed as mean ± SD (*n* = 6 per group). **(B)** Colon histopathology score, *n* = 3. Compared with normal group, ^###^ *p* < 0.001; compared with model group, ^**^ *p* < 0.01, ^***^ *p* < 0.001.

As shown in Figure 4, the mucosal state of the colonic tissues in the normal group was normal, and the intestinal tissues were structurally stable with clear staining. Conversely, in the model group, the mucosal layer of the colonic tissue was infiltrated by focal inflammatory cells, with localized submucosal edema, and exhibited a loose arrangement of connective tissue. In the simvastatin and *Usnea*_H groups, a small amount of mucosal epithelial cells was observed in the colonic tissues, and the submucosal layer was edematous. There was no inflammatory infiltration in the colon tissue in the *Usnea*_L and *Usnea*_M groups. This indicates that treatment with UEE ameliorated mucosal damage and inflammatory cell infiltration.

### 3.3 UEE decreases serum lipid levels

**FIGURE 5:**
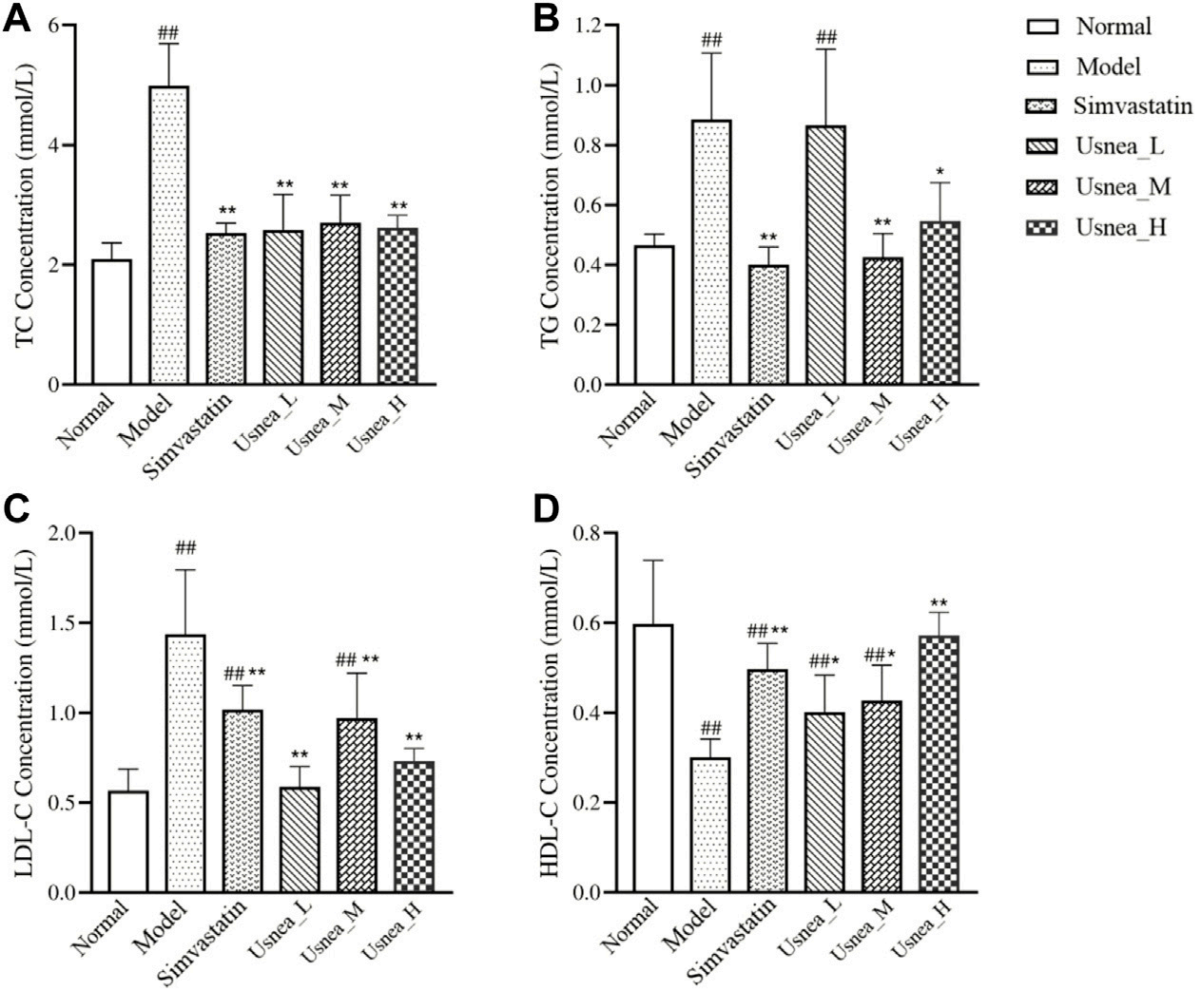
Effects of UEE on serum lipid levels in rats. **(A)** Effect of UEE on TC concentration; **(B)** effect of UEE on TG concentration; **(C)** effect of UEE on LDL-C concentration; **(D)** effect of UEE on HDL-C concentration. Results are expressed as the mean ± SD (*n* = 6 per group). Compared with the normal group, ^#^ *p* < 0.05, ^##^ *p* < 0.01; compared with the model group ^*^ *p* < 0.05, ^**^ *p* < 0.01.

After long-term consumption of a high-fat diet, TC, TG, and LDL-C levels were significantly increased and HDL-C levels were significantly decreased in AS rats compared with rats in the normal group (Figure 5). Compared with AS rats, the following changes were observed after four consecutive weeks of intervention: the simvastatin and all UEE groups had considerably lower TC levels (Figure 5A); the simvastatin, *Usnea*_M, and *Usnea*_H groups had significantly lower TG levels (Figure 5B); the simvastatin and all UEE groups had significantly lower LDL-C levels in AS rats (Figure 5C), but the LDL-C level differed between the simvastatin and *Usnea*_M groups, compared with normal rats; and the simvastatin, *Usnea*_L, *Usnea*_M, and *Usnea*_H groups had significantly higher HDL-C levels in AS rats, but HDL-C levels in the simvastatin, *Usnea*_L, and *Usnea*_M groups remained lower than those detected in the normal group (Figure 5D).

### 3.4 UEE regulates inflammatory responses linked to the intestinal flora

**FIGURE 6:**
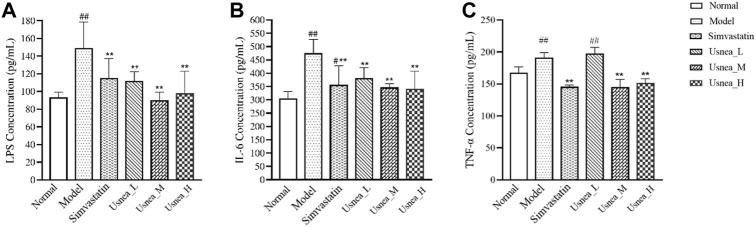
UEE affected the concentrations of LPS, IL-6, and TNF-α in the serum. **(A)** UEE decreased the concentration of LPS; **(B)** UEE inhibited the expression of IL-6; **(C)** UEE decreased the concentration of TNF-α. Results are expressed as the mean ± SD (*n* = 6 per group). Compared with the normal group, ^#^ *p* < 0.05, ^##^ *p* < 0.01; compared with the model group ^*^ *p* < 0.05, ^**^ *p* < 0.01.

As demonstrated in Figure 6, after 4 weeks of continuous modeling, the LPS levels in the model group were substantially increased compared with the levels in the normal group (*p* < 0.01). The simvastatin and all UEE groups exhibited a significant decrease in serum LPS levels (*p* < 0.01, Figure 6A). IL-6 levels were markedly elevated in the model group and were considerably higher than those in the simvastatin and all UEE groups (*p* < 0.01, Figure 6B). Additionally, TNF-α level was significantly increased in the model group. Compared with the model group, TNF-α levels were significantly decreased in the simvastatin, *Usnea*_M, and *Usnea*_H groups (*p* < 0.01, Figure 6C).

### 3.5 UEE remodels the intestinal flora of AS rats

#### 3.5.1 Analysis of the alpha diversity of the intestinal flora

**FIGURE 7:**
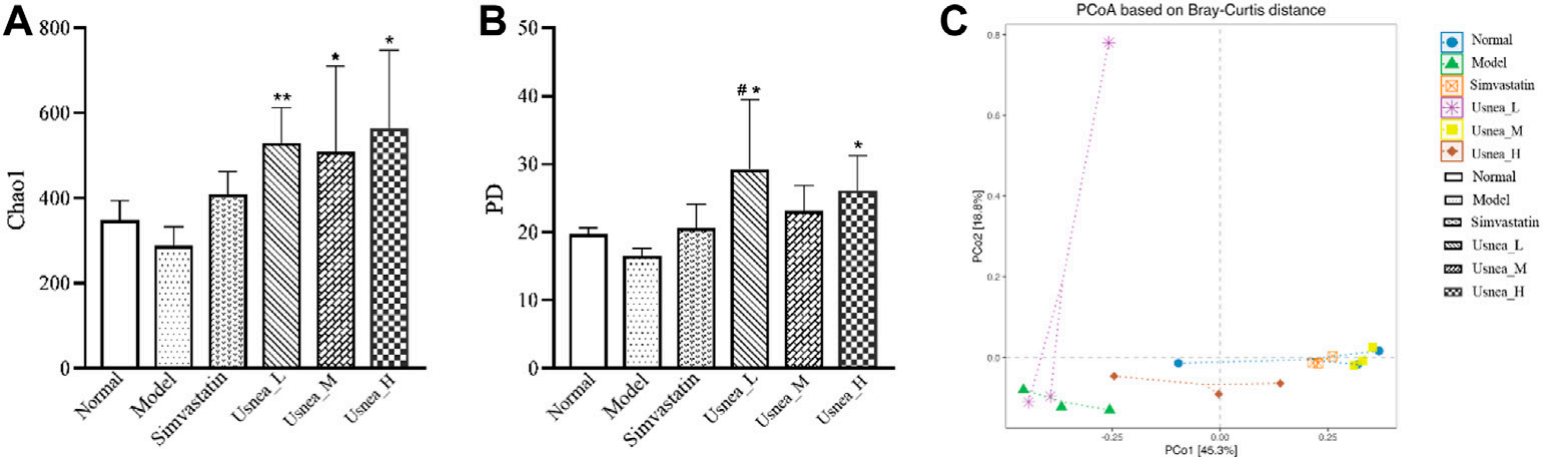
Intestinal flora alpha diversity index. **(A)** Chao1; **(B)** Faith’s phylogenetic diversity; **(C)** Beta diversity of AS rats, including PCoA. Results are expressed as the mean ± SD (*n* = 3 per group). Compared with the normal group, ^#^ *p* < 0.05; compared with the model group, ^*^ *p* < 0.05, ^**^ *p* < 0.01.

The Chao1 and Faith’s phylogenetic diversity (PD) values are the most commonly used alpha diversity indices. The Chao1 index is primarily used to determine the overall number of species, whereas Faith’s PD measures PD based on an evolutionary tree. As demonstrated in Figure 7, the low, medium, and high doses of UEE substantially enhanced Chao1 levels compared with AS rats. The normal and simvastatin groups tended to have higher Chao1 levels compared with AS rats; however, the differences were not statistically significant (Figure 7A). In terms of PD values, rats in the *Usnea*_L and *Usnea*_H groups had significantly higher PD values than rats in the AS group (Figure 7B).

#### 3.5.2 Analysis of the beta diversity of intestinal flora

In order to conduct follow-up analysis based on the calculated distance indicators between various commonly used samples, and at the same time to facilitate the observation of the degree of difference between samples and the law of difference change, principal coordinate analysis (PCoA) was used for exploration. PCoA is a common method for analyzing beta diversity. The dots show the relative position of the samples; the closer the dots are to one another, the less structural variation there is in the representative population, and *vice versa*. As shown in Figure 7C, except for the *Usnea*_L group, the distance between the treatment and model groups was farther, indicating that the simvastatin, *Usnea*_M, and *Usnea*_H treatments could alter the intestinal flora structure of AS rats, bringing it closer to that of the normal group.

#### 3.5.3 Community composition analysis of the intestinal flora

**FIGURE 8:**
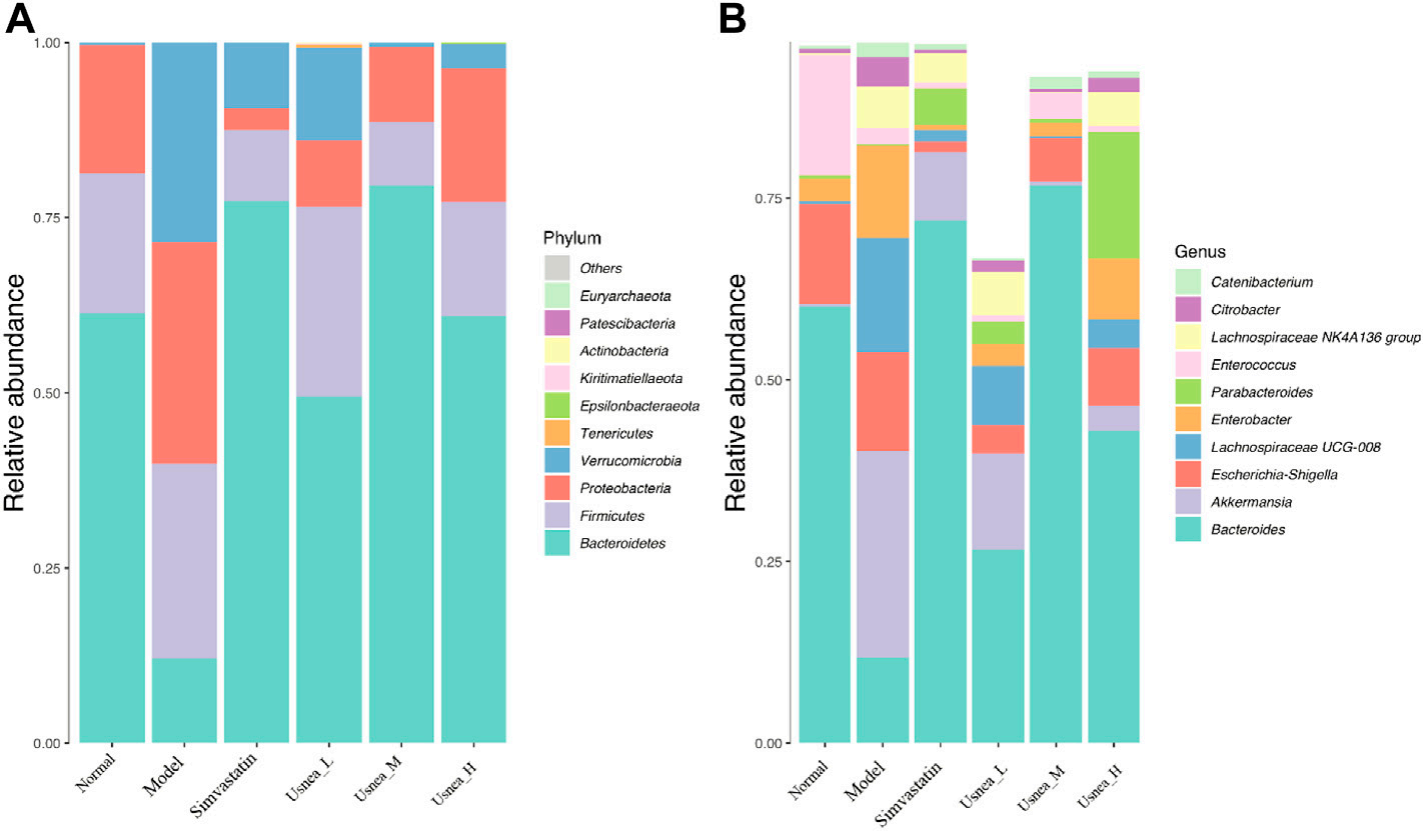
Relative abundance of organisms at the phylum and genus levels in the intestinal flora. **(A)** Relative abundance of the top 10 enteric flora at the phylum level; **(B)** relative abundance of the top 10 enteric flora at the genus level. Note: Values are expressed as mean ± SD (*n* = 3 per group). Different colors represent different enteric flora.

To determine the changes in species abundance, we selected 10 species with the highest phylum- and genus-level abundances (Figure 8). In the normal group of rats, *Bacteroidetes*, *Firmicutes*, and *Proteobacteria* represented the majority of the intestinal flora at the phylum level (Figure 8A). The relative abundance of *Verrucomicrobiota* was significantly higher (*p* < 0.01), whereas that of Bacteroidetes was considerably lower (*p* < 0.01) in the model group, although the abundance of *Firmicutes* and *Proteobacteria* did not differ significantly between the groups. Compared with the model rats, all UEE dosages decreased the relative abundance of *Verrucomicrobiota* (*p* < 0.01) while they increased that of *Bacteroidetes* (*p* < 0.01, *p* < 0.05). Additionally, the *Usnea*_L and *Usnea*_M groups had a substantially lower relative abundance of *Proteobacteria* than other groups.

At the genus level, *Bacteroides*, *Escherichia–Shigella*, and *Enterococcus* were the predominant genera of the intestinal flora in the normal group of rats (Figure 8B). The relative abundance of *Bacteroides* and *Enterococcus* was substantially lower in the model group than in the normal group (*p* < 0.01); in contrast, the abundance of *Akkermansia*, Lachnospiraceae *UCG-008*, *Enterobacter*, Lachnospiraceae *NK4A136 group*, and *Citrobacter* was significantly increased (*p* < 0.01). Compared with the model rats, the alteration of the intestinal flora in the simvastatin, *Usnea*_L, *Usnea*_M, and *Usnea*_H groups was corrected to varying degrees.

#### 3.5.4 Species analysis of community differences in intestinal flora

**FIGURE 9:**
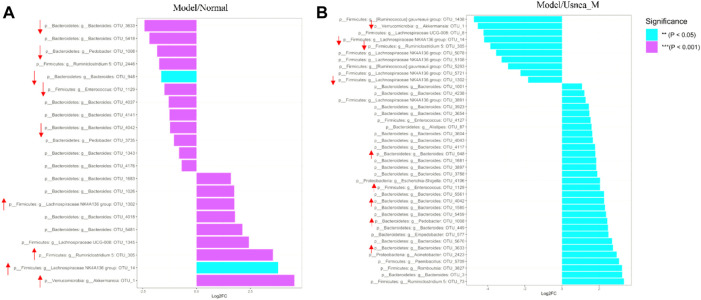
Differential analysis of the intestinal flora. **(A)** Differential intestinal flora at the phylum and genus levels between normal and model rats. **(B)** Differential intestinal flora at the phylum and genus levels between rats in the model and *Usnea*_M groups. Positive values of log_2_FC (fold change) on the horizontal axis represent upregulated species, whereas negative values represent downregulated species. Up- and downregulation pertain to rats in the model group. The vertical axis indicates the species taxonomic attributes, with three columns for the phylum, genus, and operational taxonomic unit (OTU). As the specific species of intestinal bacteria could not be determined in this study, the OTU values represent the species within the appropriate genus of intestinal bacteria. The color indicates the degree of relevance.

The metagenomeSeq method was used to search for statistically significant differences in the intestinal microbiota that were associated with AS and modulated by UEE (Paulson et al., 2013). As shown in Figure 9, seven types of intestinal genera related with AS could be divided into 21 intestinal bacteria (Figure 9A). Because of the inability to identify the exact species of intestinal bacteria in this study, the OTU values represent the species within the appropriate genus of intestinal bacteria. Compared with the normal group of rats, the relative abundance of *Bacteroides* OTU_4176, 4042, 3,735, 4141, 4037, 948, 5,418, and 3,633, *Pedobacter* OTU_3735 and 1008, *Enterococcus* OTU 1129, and *Ruminiclostridium 5* OTU_2446 was decreased in the model group. The species that exhibited an increased relative abundance were *Akkermansia* OTU_1, Lachnospiraceae *NK4A136 group* OTU_14 and 1320, *Ruminiclostridium 5* OTU_305, Lachnospiraceae *UCG-008* OTU_1345, and *Bacteroides* OTU_5418, 4018, 1026, and 1683. Notably, UEE treatment reversed the relative abundance of six groups of enteric species (Figure 9B) (i.e., *Akkermansia* OTU_1, Lachnospiraceae *NK4A136 group* OTU_14 and 1320, *Ruminiclostridium 5* OTU_305, *Bacteroides* OTU_4042, 948, and 3,633, *Pedobacter* OTU_1008, and *Enterococcus* OTU_1129).

### 3.6 UEE regulates the levels of TMAO and BAs associated with intestinal flora

**FIGURE 10:**
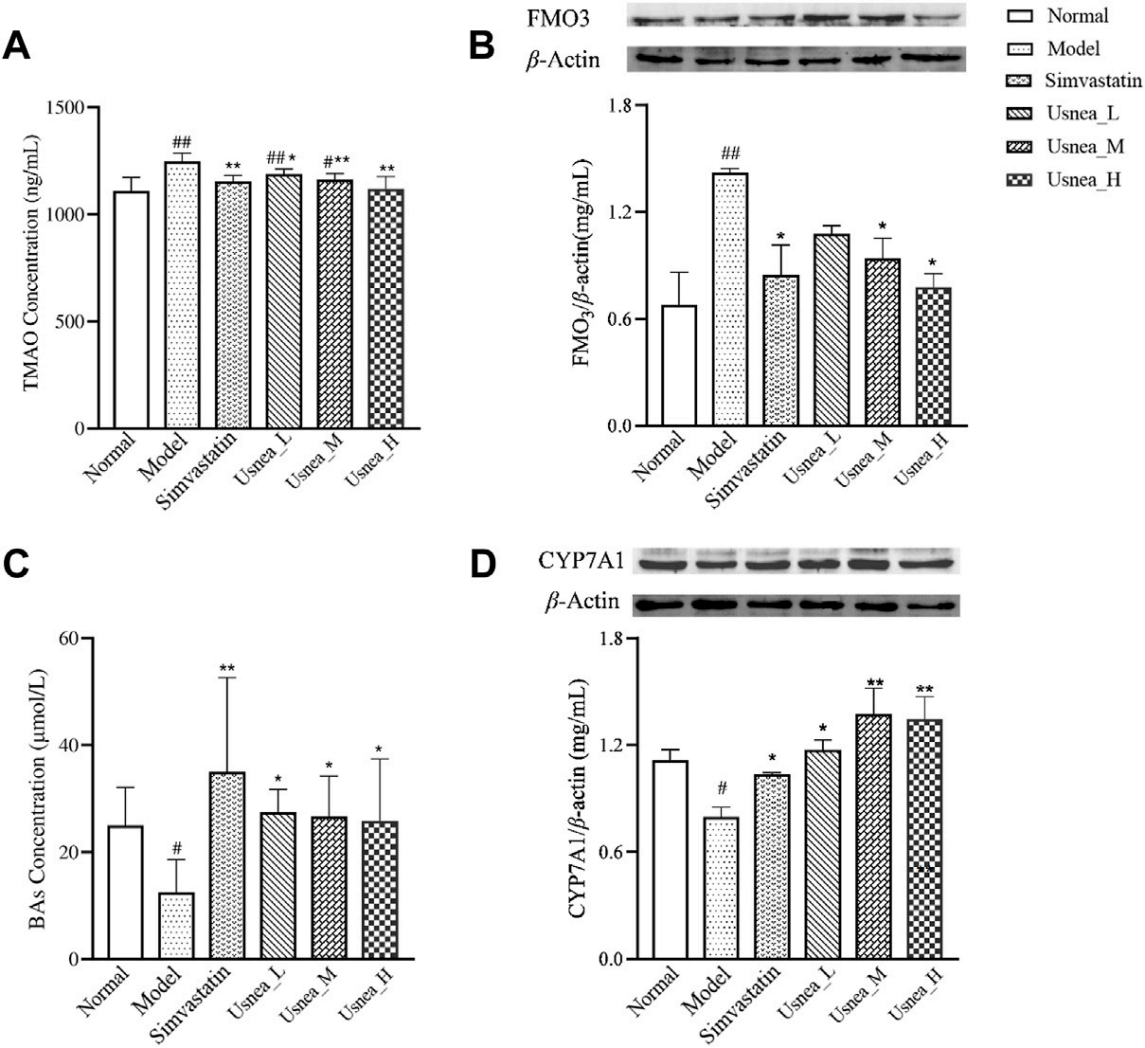
UEE affected the control of the metabolic pathways of TMAO and BAs. **(A)** UEE decreased the concentration of TMAO; **(B)** UEE inhibited the expression of FMO3; **(C)** UEE increased the concentration of BAs; and **(D)** UEE increased the expression of the CYP7A1 protein. Note: Values are expressed as mean ± SD (*n* = 6 per group). ^#^ *p <* 0.05, ^##^ *p <* 0.01 compared with the normal group; ^*^ *p <* 0.05, ^**^ *p <* 0.01 compared with the model group.

As demonstrated in Figure 10A, the plasma TMAO levels were substantially higher in the model group than in the normal group (*p* < 0.01). After 4 weeks of treatment, UEE and simvastatin effectively decreased plasma TMAO levels in AS model rats (*p* < 0.01 or *p* < 0.05).

As shown in Figure 10B, the protein expression level of FMO3 in the liver of rats in the model group was substantially elevated (*p* < 0.01). After four consecutive weeks of treatment, the protein expression of FMO3 in the rat liver was considerably reduced in the simvastatin, *Usnea*_M, and *Usnea*_H groups (*p* < 0.01 or *p* < 0.05).

As indicated in Figure 10C, the serum level of BAs in the model group was considerably decreased (*p* < 0.05). After four consecutive weeks of treatment, all UEE and the simvastatin groups exhibited considerably elevated serum levels of BAs (*p* < 0.01 or *p* < 0.05).

The protein expression of CYP7A1 in the liver of rats in the model group was the lowest among the six groups and differed significantly from that recorded in the normal group (*p* < 0.05), as shown in Figure 10D. After 4 weeks of treatment, the protein expression levels of CYP7A1 in the rat liver were considerably elevated in all UEE and simvastatin groups (*p* < 0.05).

### 3.7 UEE regulates intestinal permeability

**FIGURE 11:**
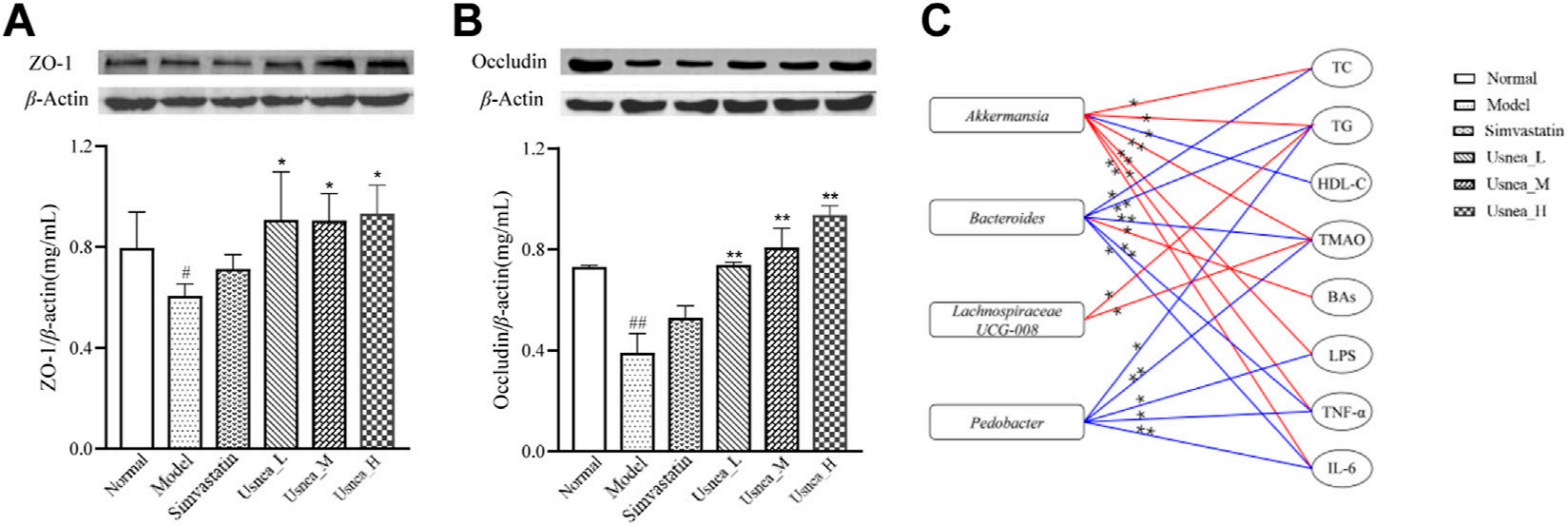
UEE affected the concentrations of occludin and ZO-1. **(A)** UEE increased the expression of ZO-1; **(B)** UEE increased the concentration of occludin; and **(C)** potential correlation analysis between the intestinal flora associated with AS and pharmacokinetic markers. Rectangles represent the intestinal flora related to AS; circles denote pharmacokinetic markers; red indicates a positive association, whereas blue indicates a negative correlation. Results are expressed as the mean ± SD (*n* = 3 per group). ^*^ *p* < 0.05, ^**^ *p* < 0.01.

To further investigate intestinal permeability, the protein expression of ZO-1 and occludin was evaluated (Figures 11A,B). ZO-1 and occludin levels were considerably lower in the model group than in the normal group (*p* < 0.01 or *p* < 0.05). Except for the simvastatin group, the expression of ZO-1 and occludin was significantly upregulated in the *Usnea*_L, *Usnea*_M, and *Usnea*_H groups compared with the model group.

### 3.8 GM-PD correlation analysis

At the genus level, Spearman correlation predictions further revealed potential associations between AS-associated enteric genera and pharmacodynamic indicators (TC, TG, etc.). As shown in Figure 11C, *Akkermansia* may be positively correlated with TC (*r* = 0.52, *p* = 0.027), TG (*r* = 0.553, *p* = 0.017), TMAO (*r* = 0.779, *p* < 0.01), LPS (*r* = 0.77, *p* < 0.01), TNF-α (*r* = 0.56, *p* = 0.016), and IL-6 (*r* = 0.726, *p* = 0.001) and negatively correlated with HDL-C (*r* = −0.526, *p* = 0.025). Notably, *Bacteroides* was positively correlated with TC (*r* = −0.538, *p* = 0.021), TG (*r* = −0.782, *p* < 0.01), TMAO (*r* = −0.675, *p* = 0.009), TNF-α (*r* = −0.675, *p* = 0.002), IL-6 (*r* = −0.517, *p* = 0.028), and BAs (*r* = 0.523, *p* = 0.026).

## 4 Discussion

Our previous studies have shown that UEE plays the role in regulation of lipid metabolism (Zhu et al., 2017; Zhao et al., 2019). The present research further demonstrated that anti-atherosclerosis effect of UEE and its mechanism.

After 14 weeks of ingestion of a high-fat diet, the model group exhibited calcium deposition and lipid degeneration in the aortic tissues as well as significant dysbiosis of the intestinal flora, indicating that an AS model in rats was effectively created *via* the consumption of high-fat diet combined with an intraperitoneal injection of VD_3_. The levels of TC, TGs, HDL-C, and LDL-C are commonly measured during the clinical examination of blood lipid levels. Rats with AS exhibited an abnormal increase in TC, TG, and LDL-C levels and a substantial decrease in HDL-C levels. In this study, UEE considerably decreased the serum levels of TC, TGs, and LDL-C and significantly increased the level of HDL-C in AS model rats, indicating that UEE has a positive regulatory effect on the body’s lipid metabolism.

The intestinal flora can induce the onset and development of AS either directly or indirectly. Among the underlying pathways, abnormalities of the intestinal flora can cause inflammation and increase the permeability of the intestinal mucosa, resulting in the entry of intestinal-derived endotoxins, bacteria, and their metabolites into the bloodstream, consequently causing damage to the organism (Tang et al., 2017). Therefore, the amelioration of the dysbiosis of the composition and function of intestinal flora has considerable value in the prevention and treatment of AS and has become a new focus of research (Nagpal et al., 2016). In this study, we discovered that AS model rats had a significantly different intestinal community structure compared with that in normal rats; however, after 4 weeks of continuous intervention with UEE, the intestinal flora disorder observed in AS model rats was significantly corrected at the phylum and genus levels and converged with that of the normal group. At the phylum level, UEE significantly reduced the amount of Proteobacteria, which are usually considered as pathogens, and increased the amount of *Bacteroides* commonly considered as beneficial bacteria. At the genus level, *Bacteroides*, *Akkermansia*, *Enterococcus*, Lachnospiraceae *UCG-008*, Lachnospiraceae *NK4A136 group*, *Ruminiclostridium 5*, and *Pedobacter* were identified as the intestinal genera related to AS. Among the community genera in each group of rats, we discovered that the relative abundance of *Bacteroides* was much lower in model rats than in normal rats, but it was markedly increased in AS rats treated with UEE. Similar to our findings, Yin et al. showed a decrease in the relative abundance of *Bacteroides* in patients with atherosclerotic ischemic stroke and transient ischemic attack (Yin et al., 2015). Gavage with live *Bacteroides vulgatus* and *Bacteroides dorei* inhibited the development of atherosclerotic lesions in AS-prone mice (Yoshida et al., 2018). Moreover, as demonstrated in a previous study, the relative proportion of *Akkermansia* in the atherosclerotic colonic microbiota was considerably higher in rats that were administered a high-fat diet than in normal rats (Liu et al., 2020). Similar results were reported for the fluctuation in the relative abundance of *Akkermansia*; however, it is interesting that the bulk of recent investigations support the opposite conclusion (Lyu et al., 2017). Thus, additional research is required to determine the variance of *Akkermansia* in AS. *Ruminiclostridium 5* and Lachnospiraceae *NK4A136* are related to inflammatory bowel disease, and Lachnospiraceae *NK4A136* may also affect the expression of ZO-1 and occludin protein, thereby affecting the intestinal barrier (Shao et al., 2020; Zhang et al., 2022). *Enterococcus*, as a probiotic, may contribute to the transport of cholesterol, and may have a cholesterol lowering effect on hypercholesterolemic mice (Cavallini et al., 2009; Zhu et al., 2019); It was also found that *Enterococcus* was negatively correlated with TC and non-high-density lipoprotein cholesterol levels, and positively correlated with HDL-C (Cavallini et al., 2011). These studies are consistent with our findings. As a genus related to obesity, *Pedobacter* has not been associated with AS in any previous studies (Palmas et al., 2021).

To explore the possible mechanism underlying the effects of UEE based on the modulation of intestinal flora to improve AS, we examined whether UEE could downregulate TMAO, attenuate its inhibitory effect on CYP7A1 expression, increase the conversion of cholesterol to BAs, and promote the metabolism of body lipids. We also tested whether UEE could improve intestinal permeability, limit intestinal-derived LPS absorption into the blood, and prevent the development of inflammatory response.

TMAO can enhance the development and progression of AS *via* several pathways and is a significant predictor of AS risk (Tang et al., 2013). In the present study, the long-term consumption of a high-fat diet increased the protein expression of FMO3 in the liver of rats in the model group, which resulted in a significant increase in the plasma level of TMAO. TMAO supports the development and progression of AS *via* several mechanisms, and an increase in TMAO levels is associated with the progression of AS lesions (Din et al., 2019). After 4 weeks of intervention, UEE substantially downregulated the protein expression of FMO3, reduced TMAO levels in the plasma of model rats, and decreased the AS load in model rats. The formation of BAs is the primary mechanism of cholesterol breakdown. The addition of adequate amounts of BAs to a high-fat diet is helpful in decreasing LDL levels and plaque formation in AS, according to a previous study (Araki et al., 2005). BAs are the ultimate cholesterol metabolites, and the body primarily synthesizes BAs *via* the classical pathway to accelerate the conversion and breakdown of cholesterol in hepatocytes. CYP7A1 is a critical rate-limiting enzyme in the traditional BA production pathway, and TMAO can downregulate the activity of CYP7A1 (Pikuleva, 2008). After 4 weeks of UEE intervention, the protein expression of CYP7A1 tended to increase and the level of serum BAs was significantly increased, indicating that UEE upregulated CYP7A1 expression in the liver, thereby increasing lipid metabolism, and increased the level of BAs, thereby decreasing blood lipid levels. Therefore, our data suggest that UEE can promote lipid metabolism and reduce calcium deposition in rats by reducing the protein expression of FMO3 in liver tissues and plasma levels of TMAO, increasing the protein expression of CYP7A1, increasing the levels of serum BAs, accelerating cholesterol metabolism, and reducing lipid levels to ameliorate AS lesions.

The long-term consumption of a high-fat diet induces a local inflammatory response in the intestine, resulting in impaired intestinal barrier function and increased permeability; thus, LPS and microorganisms can enter the body circulation through the damaged intestinal epithelium, further inducing inflammatory response or a disturbance in lipid metabolism, thereby initiating or aggravating AS lesions (Ostos et al., 2002). ZO-1 and occludin are key proteins in the intestinal mucosal barrier and significant markers of tight junction integrity (Gilani et al., 2018). UEE significantly increased the protein expression of ZO-1 and occludin in the colon tissue of AS model rats and improved the intestinal mucosal barrier function after 4 weeks of continuous intervention. An intact intestinal barrier is the most effective method of preventing the entry of intestinal LPS, bacteria, and their metabolites into the circulation. LPS acts as an inflammatory irritant by promoting the development and release of various inflammatory factors, such as TNF-α and IL-6, through the modulation of the NF-κB and MAPK pathways (Valentinis et al., 2005). In our experiments, we discovered a significant increase in LPS levels in the serum of rats with AS compared with the normal group. In previous studies on AS, excessive injections of IL-6 resulted in the creation of fatty plaques, and high levels of IL-6 increased the instability of plaques. IL-6 has a regulatory effect on TNF-α, which contributes to the generation and release of IL-6 during inflammatory response (Choussat et al., 2000). TNF-α increases LDL levels and decreases HDL levels, thereby increasing the risk of lipid metabolism disorder (Rizvi, 2007). The administration of UEE to rats with AS dramatically decreased blood LPS, TNF-α, and IL-6 levels and inhibited inflammatory response in AS model rats. UEE enhanced the intestinal mucosal barrier function of the colon tissue by upregulating the protein expression of tight junction-related proteins ZO-1 and occludin, reduced the absorption of intestinal-derived LPS into the blood, and significantly decreased serum LPS levels in AS model rats, consequently reducing the inflammatory response induced by LPS and the AS load *via* various pathways. At the same time, many studies found that the increase of beneficial bacteria, such as *Bacteroides* and *Enterococcus*, would promote the expression of tight junction protein occludin and ZO-1, and accompanied by the reduction of harmful bacteria of Lachnospiraceae *NK4A136* (Yan et al., 2018; Li et al., 2022). These studies are consistent with our findings. Spearman’s rank correlation revealed significant positive or negative associations between *Bacteroides*, *Akkermansia*, and AS rats with respect to lipid metabolism, TMAO and BA metabolism, and inflammatory response. *Bacteroides* are directly involved in the production of short-chain fatty acids to preserve the integrity of the intestinal barrier and minimize intestinal inflammation (Shen et al., 2017). They are also indirectly implicated in the production of TMAO and BAs (Lyu et al., 2017). Changes in the abundance of *Bacteroides* have tremendous potential for predicting coronary artery disease (Derrien et al., 2017). Liu et al. (2020) found that TG and LDL levels were negatively correlated with *Akkermansia*. Overall, these findings indicate that UEE can improve AS by increasing the abundance of *Bacteroides*, *Pedobacter*, and *Enterococcus* and decreasing that of *Akkermansia*, Lachnospiraceae *NK4A136*, and *Ruminiclostridium 5*.

## 5 Conclusion

In AS rat models, we found that UEE remodeled the intestinal flora balance, corrected lipid metabolic abnormalities, and controlled inflammatory responses. Additionally, it markedly reduced calcium deposition in the aorta and effectively alleviated AS. The mechanism of action of UEE may involve an increase in the abundance of *Bacteroides*, *Pedobacter*, and *Enterococcus* and a decrease in that of *Akkermansia*, Lachnospiraceae *NK4A136*, and *Ruminiclostridium 5*, thereby decreasing the content of the intestinal flora metabolite TMAO, increasing the levels of BAs, and promoting lipid metabolism while enhancing the intestinal mucosal barrier function and reducing the absorption of intestinal LPS. Furthermore, UEE decreased inflammatory responses, ultimately reducing calcium deposition in the aorta. Our findings provide additional theoretical support for the ability of *Usnea* to alleviate AS by targeting the intestinal flora.

## Data Availability

The original contributions presented in the study are included in the article/supplementary material, further inquiries can be directed to the corresponding authors.

## Abbreviations

ANOVA, One-way analysis of variance; AS, Atherosclerosis; BAs, Bile acids; CYP7A1, Cholesterol 7α-hydroxylase; ECL, Electrogenerated chemiluminescence; FMO3, Flavin monooxygenase 3; gDNA, genomic DNA, HDL-C; High-density lipoprotein cholesterol, HE; Hematoxylin and eosin, HRP; Horseradish peroxidase, IL-6; Interleukin-6, LDL-C; Low-density lipoprotein cholesterol, LPS; Lipopolysaccharide, LSD; Least significant difference, OTU; Operational taxonomic uni, PBS; Phosphate-buffered saline, PVDF; Polyvinylidene fluoride, TC; Total cholesterol, TG; Triglycerides, TMAO; Trimethylamine oxide, TNF-α; Tumor necrosis factor-α, UEE; *Usnea* ethanol extract, VD_3_; Vitamin D_3_.
